# Badger—an accessible genome exploration environment

**DOI:** 10.1093/bioinformatics/btt466

**Published:** 2013-08-11

**Authors:** Ben Elsworth, Martin Jones, Mark Blaxter

**Affiliations:** Institute of Evolutionary Biology, University of Edinburgh, West Mains Road, Edinburgh EH9 3JT, UK

## Abstract

**Summary:** High-quality draft genomes are now easy to generate, as sequencing and assembly costs have dropped dramatically. However, building a user-friendly searchable Web site and database for a newly annotated genome is not straightforward. Here we present Badger, a lightweight and easy-to-install genome exploration environment designed for next generation non-model organism genomes.

**Availability:** Badger is released under the GPL and is available at http://badger.bio.ed.ac.uk/. We show two working examples: (i) a test dataset included with the source code, and (ii) a collection of four filarial nematode genomes.

**Contact:** mark.blaxter@ed.ac.uk

## 1 INTRODUCTION

High-throughput sequencing has revolutionized genome sequencing. New sequencing technologies and improved computational tools mean that a high quality draft eukaryotic genome can be sequenced and assembled in days, on a budget accessible to most researchers. This has produced an explosion of genome projects, an increasing number of which involve multiple species or strains.

Genome data and annotation must be made accessible to collaborators via a restricted environment and to the wider research community following publication. Several genome exploration environments (GEEs) exist, including ENSEMBL (Flicek *et al.*, 2013) and National Center for Biotechnology Information (NCBI) Genomes (http://www.ncbi.nlm.nih.gov), but these do not allow real-time updates or restrictions on data access. ENSEMBL can be used as a stand-alone GEE, as can projects like GeneDB (Logan-Klumpler *et al.*, 2012), but these require skills not available in groups new to genome informatics. GEEs that use the Generic Model Organism Database (GMOD) Chado database model (Mungall *et al.*, 2007), including Tripal (Ficklin *et al.*, 2011) and Chado on Rails (http://gmod.org/wiki/Chado_on_Rails), are similar to ENSEMBL in scope. Tripal is the most mature GMOD GEE and is widely used, but requires a dedicated software engineer to develop and design each genome instance.

We here present Badger, an easy-to-install GEE for genome scientists who need to produce a web-accessible portal for new data. A single installation of Badger can contain data from multiple species, and each species can have multiple genome drafts and gene sets.

## 2 FEATURES

A Badger site is managed by an administrator and has independent visibility settings (open or restricted by login) for all major sections. The *home page* for a Badger instance is fully customizable using a built-in text editor. It can be used to give project background and contains a blog-style news feed for up-to-date commentary. An information button is present on all pages, providing a guide to the site and page-specific information.

The *species overview* page introduces the species included in the GEE and their relationships, including an optional phylogram. After selecting a species, the user selects a genome assembly version and a gene annotation version (multiple versions can coexist). These are displayed on the *genome overview* page, which displays *summary* metrics, including an interactive chart of metrics for each individual scaffold or contig (collectively referred to as genome objects). Selecting a genome object on the chart links to a page with detailed information. For genome objects with location data (contigs, scaffolds, genes and transcripts), an embedded GBrowse (Stein *et al.*, 2002) instance can be used to display it in genomic context. A *search* tab offers four ways of searching: (i) a broad search of all annotations and relevant publications, (ii) a restricted search of particular types of annotation (BLAST similarity, domain annotation, etc.), (iii) search for genes with a particular identifier and (iv) detailed searching of publication abstracts. Where appropriate, search results are presented as a table that allows sorting and filtering. All genome, transcript and protein sequence data can be searched using BLAST (Camacho *et al.*, 2009), with results presented in plain text (for BLAST output) or FASTA (for matching sequences) format.

Individual *gene* pages offer a rich summary of metrics and annotation. Orthology information is a powerful way of exploring cross-species similarities, or drilling down into a biological component of interest. Optionally, genes can be grouped into putative ortholog sets using OrthoMCL (Li *et al.*, 2003). Orthology assignment is available on the *gene* page, and ortholog groups can also be identified by direct annotation search or by membership criteria (for example 1:1:1 … orthology). The *ortholog overview* page contains an interactive chart showing the size, frequency and proportion of members in each dataset, along with alignments.

## 3 IMPLEMENTATION

Badger is built using the Grails (http://grails.org) web application framework and uses the dynamic language Groovy, which runs on the Java Virtual Machine. Badger takes advantage of Grails’ embedded web server and uses plug-ins to provide caching and access control. Genome and annotation data are stored in only 15 custom domain classes, making the code base easy to understand and extend. Badger uses PostGresSQL (http://www.postgresql.org/) as a persistent data store.

Badger was developed on Linux and has been deployed on both Ubuntu (12.04) and Centos (6.3) distributions. Hardware requirements will vary with the size of the dataset; we recommend a minimum of 4 GB random access memory and 2 central processing unit cores. Badger requires Grails (v2.1+), Java Development Kit (v1.6+), PostGreSQL (v8.4+) and BLAST+ (v2.2.26+). Detailed installation instructions are available at https://badger.bio.ed.ac.uk. Optionally, OrthoMCL (v2.0.4+) is required for clustering, Muscle (v3.8.31+) (Edgar, 2004) for ortholog group alignment, InterProScan (v4+) (Quevillon *et al.*, 2005) for domain identification and GBrowse for genome browser delivery.

**Fig. 1.**
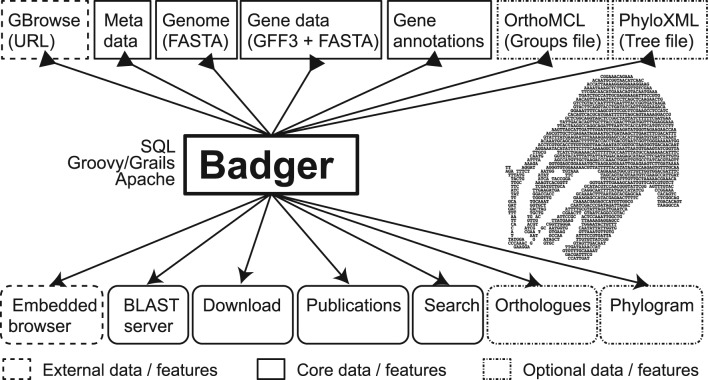
Data flow in Badger. Data requirements are split into three groups: core, optional and external. Using core data alone results in a site with BLAST server, download facility, publication database and extensive search options. If provided, an external Gbrowse instance can be embedded. Optional data from OrthoMCL and PhyloXML (Han and Zmasek, 2009) files can provide powerful gene contextualization and phylogenetic visualization

Loading data into a Badger instance is straightforward (Fig. 1). First, metadata are added, including species information and sequence and annotation data file paths. The minimum data requirement is a collection of contigs in FASTA format, which will generate a basic BLAST server, a genome overview page and a sequence download tool. The full potential of the GEE is achieved with annotated gene sets. Badger accepts gene set data (GFF3 format) with corresponding transcript and protein sequences (FASTA format), and also data in BLAST XML output, InterProScan raw output and Tab Separated Value (TSV) custom annotation files. TSV import allows custom annotation types to be added without editing underlying code. Ortholog group information should be provided in OrthoMCL format. New species can be added rapidly by taking advantage of existing annotation. Badger can import data from external resources and FASTA format genome and gene sequences. External data within a GBrowse instance can also be embedded.

Data files can be uploaded, edited and updated individually through the administrator interface or *en masse* using a script (an example is provided in the source code). Once data files are uploaded and metadata entered, Badger parses input files, loads data into the PostgreSQL database, sources publications for the species from NCBI PubMed, creates BLAST databases, compresses FASTA files for download, generates a phylogenetic tree and catalogs ortholog data. Publication data are updated weekly. The whole process can be trivially rerun at any time to allow the inclusion of new data. Badger data upload is fast. For computationally expensive data in overview pages, Badger makes extensive use of caching to ensure that the interface remains responsive. For full-text searching of annotations, Badger uses PostgreSQLs full-text indexing, allowing it to search around 0.5 million annotations in a few seconds. The test dataset (100 scaffolds, 289 genes and 6615 annotations) takes <5 min, whereas the four-species filarial nematode dataset (50 000 scaffolds, 50 000 genes and 700 000 annotations) takes <24 h, to recreate on a minimum specification machine.

## 4 CONCLUSION

Badger is a lightweight GEE with a gene- and annotation-centric approach capable of storing, searching and visualizing diverse genomic data. Installation is simple, and a fully functional instance can be created quickly, even by novice users. Although not a substitute for data submission to databases of record, Badger is a customizable environment for public and collaborative display of gene-centred genomic information, and will aid in preparation for publication and submission.
